# Sociocultural determinants of anticipated oral cholera vaccine acceptance in three African settings: a meta-analytic approach

**DOI:** 10.1186/s12889-016-2710-0

**Published:** 2016-01-14

**Authors:** Neisha Sundaram, Christian Schaetti, Sonja Merten, Christian Schindler, Said M. Ali, Erick O. Nyambedha, Bruno Lapika, Claire-Lise Chaignat, Raymond Hutubessy, Mitchell G. Weiss

**Affiliations:** 1 Department of Epidemiology and Public Health, Swiss Tropical and Public Health Institute, Socinstrasse 57, 4002 Basel, Switzerland; 2 University of Basel, Petersplatz 1, 4003 Basel, Switzerland; 3 Saw Swee Hock School of Public Health, National University of Singapore, 12 Science Drive 2, Singapore, 117549 Singapore; 4 Public Health Laboratory Ivo de Carneri, Chake-Chake, Pemba, Zanzibar United Republic of Tanzania; 5 Department of Sociology and Anthropology, Maseno University, Private Bag, Maseno, Kenya; 6 Department of Anthropology, University of Kinshasa, Kinshasa, Democratic Republic of Congo; 7 Global Task Force on Cholera Control, World Health Organization, 20, Avenue Appia, 1211 Geneva 27, Switzerland; 8 Initiative for Vaccine Research, World Health Organization, 20, Avenue Appia, 1211 Geneva 27, Switzerland

**Keywords:** Vaccine acceptance, Cholera vaccine, Social determinants, Cultural epidemiology, Meta-analysis, Africa

## Abstract

**Background:**

Controlling cholera remains a significant challenge in Sub-Saharan Africa. In areas where access to safe water and sanitation are limited, oral cholera vaccine (OCV) can save lives. Establishment of a global stockpile for OCV reflects increasing priority for use of cholera vaccines in endemic settings. Community acceptance of vaccines, however, is critical and sociocultural features of acceptance require attention for effective implementation. This study identifies and compares sociocultural determinants of anticipated OCV acceptance across populations in Southeastern Democratic Republic of Congo, Western Kenya and Zanzibar.

**Methods:**

Cross-sectional studies were conducted using similar but locally-adapted semistructured interviews among 1095 respondents in three African settings. Logistic regression models identified sociocultural determinants of OCV acceptance from these studies in endemic areas of Southeastern Democratic Republic of Congo (SE-DRC), Western Kenya (W-Kenya) and Zanzibar. Meta-analytic techniques highlighted common and distinctive determinants in the three settings.

**Results:**

Anticipated OCV acceptance was high in all settings. More than 93 % of community respondents overall indicated interest in a no-cost vaccine. Higher anticipated acceptance was observed in areas with less access to public health facilities. In all settings awareness of cholera prevention methods (safe food consumption and garbage disposal) and relating ingestion to cholera causation were associated with greater acceptance. Higher age, larger households, lack of education, social vulnerability and knowledge of oral rehydration solution for self-treatment were negatively associated with anticipated OCV acceptance. Setting-specific determinants of acceptance included reporting a reliable income (W-Kenya and Zanzibar, not SE-DRC). In SE-DRC, intention to purchase an OCV appeared unrelated to ability to pay. Rural residents were less likely than urban counterparts to accept an OCV in W-Kenya, but more likely in Zanzibar. Prayer as a form of self-treatment was associated with vaccine acceptance in SE-DRC and W-Kenya, but not in Zanzibar.

**Conclusions:**

These cholera-endemic African communities are especially interested in no-cost OCVs. Health education and attention to local social and cultural features of cholera and vaccines would likely increase vaccine coverage. High demand and absence of insurmountable sociocultural barriers to vaccination with OCVs indicate potential for mass vaccination in planning for comprehensive control or elimination.

## Background

Cholera results from ingesting pathogenic strains of the bacterium *Vibrio cholerae* in contaminated water or food [1]. Although cholera should not be fatal, if untreated, case-fatality rates (CFR) for severe cholera may be as high as 50 % [2]. An estimated 1.4 billion people are at risk for cholera in endemic countries [3]. Controlling cholera remains a significant challenge in Sub-Saharan Africa. Access to safe water and sanitation remain low in the region, about 61 and 30 %, respectively [4]. Needed development requires major investments in infrastructure that proceed very slowly.

In the interim, oral cholera vaccines (OCVs) can save lives in epidemics and in endemic areas. The World Health Organization (WHO) recommends OCVs as a short-term control strategy for high-risk populations to complement long-term water and sanitation improvements [5]. Two safe OCVs—Shanchol™, with a protective efficacy of 66 % [6], and Dukoral®, with 79 % direct protection [7]—are currently available for international use. Efficacy is not enough, however, for vaccines to be effective. People must also be willing to accept them. Local social and cultural ideas about illness, vaccines and community preferences are critical considerations. Past programme experience provides valuable lessons that underscore the priority of social and cultural aspects of vaccine acceptance and effective vaccine action [8–11]. A recent review of vaccine hesitancy suggests community effectiveness may depend on particular features of setting, health problem and vaccine [12].

Making the benefits of immunization, including new and underutilized vaccines, available to all regardless of where they are born, who they are, or where they live is a vision of the Decade of Vaccines (2011–2020) [13]. In 2012, the World Health Assembly approved the Global Vaccine Action Plan as a framework to achieve this vision and a strategic objective of the plan emphasised the importance of understanding community demand and trust in vaccines. The decision made by Gavi, the Vaccine Alliance (Gavi) to contribute to a global stockpile for OCVs during 2014–2018 reflects increasing priority for use of cholera vaccines in endemic settings [14]. However, not enough is known about community acceptance of OCVs, especially across populations, and this information is critical for effective vaccine implementation. Furthermore, although some sociocultural features may have consistent effects on across settings, others are specific to particular local settings. Systematic comparison of local studies clarifies consistent and distinctive effects of sociocultural factors on vaccine acceptance that may not be apparent from findings of the local studies. This interest motivated the comparative analysis reported here.

This report presents findings from comparison of sociocultural determinants of anticipated OCV acceptance across the three settings in Sub-Saharan Africa: Southeastern Democratic Republic of Congo (SE-DRC), Western Kenya (W-Kenya) and Zanzibar. Studies of sociocultural aspects of cholera and determinants of anticipated OCV acceptance were undertaken in three cholera-endemic settings in Africa [15–17]. A comparison of sociocultural features of cholera illness experience and meaning have been reported by Schaetti and colleagues [18]. Common and distinctive sociocultural determinants of anticipated OCV acceptance that may affect uptake and effectiveness of OCVs in cholera-endemic areas of three countries in Sub-Saharan Africa are presented in this cross-setting analysis. Knowledge of comparative community interest and setting-specific determinants of OCV acceptance, gleaned from cross-cultural study, can guide policy for effective implementation of OCV. With the development of a global stockpile, findings to help guide use of OCVs are likely to be timely.

## Methods

### Study setting

In SE-DRC, the study was conducted in Kasenga district of Katanga province. In W-Kenya, it was conducted in Kisumu and Siaya districts of Nyanza province, located on the banks of Lake Victoria. In Zanzibar, a semi-autonomous part of the United Republic of Tanzania, study sites were located on Unguja and Pemba Islands. Study design in the three settings—SE-DRC, W-Kenya and Zanzibar—were very similar. Zanzibar is the only one of the three settings where a mass OCV vaccination was implemented [7], but study data analysed here were collected before the mass vaccination. Rural and urban (or peri-urban) sites were included in each of the settings.

In the years 2008 and 2009 a total of 53,049 cholera cases were reported to the WHO from DRC, 14,516 cases were reported from Kenya and 10,611 cases for Tanzania [19, 20]. Reported cholera cases are estimated to represent only a fraction of the actual cases due to substantial underreporting [21, 22]. All three settings lack universal access to safe water and sanitation.

### Individual study design and instruments

Three cross-sectional studies among adults in the general population were conducted. Similar, but locally adapted, semi-structured EMIC (Explanatory Model Interview Catalogue) interviews [23] that collected quantitative and narrative data were used at each setting. Interviews were translated into Kiswahili in all three settings and additionally into Dholuo for W-Kenya and CiBemba for SE-DRC. While interview questions were nearly identical in all settings, categories for coding were adapted based on pilot interviews, discussions with local public health professionals and ethnographic studies in each of the settings. Interview questions enquired about sociocultural concepts such as patterns of distress, perceived causes, help-seeking and methods of prevention associated with a cholera-like illness (presented to respondents using a clinical vignette describing a person with cardinal cholera symptoms) based on a cultural epidemiological framework [24]. Cultural epidemiology integrates the local validity of anthropology with the explanatory power of epidemiology through a mix of qualitative and quantitative research methods. Sociodemographic data and respondent ideas about vulnerability to the illness and associated stigma were also collected.

The instruments also included an assessment of respondents’ willingness to accept OCVs at different prices: ‘free’ as in the case of many mass vaccination campaigns; ‘low price’, approximately USD 1; ‘medium price’, USD 4–5 and ‘high price’, USD 8–11. The differing price levels were included to assess relative priority for OCVs in these communities. Four separate questions were posed to respondents for each of the price levels as follows: “If a vaccine that you swallow becomes available to prevent cholera, would you take it if it was made available [without charge/at price stated in local currency]”. Responses were recorded on a four -point Likert scale: ‘yes’, ‘possibly’, ‘uncertain’ and ‘no’. OCV prices were stated in the near-equivalent local currency as price per vaccine course. Overall, the instruments enabled assessment of locally-valid distributions of community ideas regarding cholera experience, meaning and behaviour, in the absence of an outbreak, and determined anticipated acceptance of OCVs in all three study settings. The EMIC interviews used in each of the settings have been presented by Schaetti et al*.* [18].

Interviews were revised after pilot-testing. Men and women from the general population, above 18 years of age, and physically and mentally able to participate in an interview that lasted approximately 45 min to one hour were included.

### Sampling and data collection

A minimum sample size of 328 was required in each of the study settings to enable cross-site comparisons with 95 % significance and 80 % power. Selection of households for interview in Zanzibar consisted of a simple random sample at the peri-urban and rural sites, drawn from geographic information system data and census information, respectively. In W-Kenya and SE-DRC, where only estimates of population size were available, sampling was based on household lists obtained through community health workers or every n^th^ household was selected systematically based on a random walk method.

Interviews were conducted by locally-recruited interviewers fluent in the local language and English. They received extensive training in sampling procedures, obtaining informed consent and interviewing in workshops prior to onset of the main study. Data collection proceeded from June through August 2008 in Zanzibar, March through May 2010 in Kenya, and August through September 2010 in SE-DRC. Further details on sampling and data collection are provided elsewhere [15–17, 25].

### Data management and approach to analysis

Quantitative data were double entered using EpiInfo software version 3.5.1 (Centers for Disease Control and Prevention, USA).

#### Explanatory variables

In each setting, prominence means were calculated for sociocultural variables (e.g., categories of distress and perceived causes) depending on how they were reported. A category reported spontaneously by the respondent in response to an open question received a higher prominence (value = 2) than responses provided only on probing (value = 1); if a category was identified as most important among all categories, a value of 3 was added. The cumulative prominence by respondent (range 0–5) was used to calculate mean prominence for each category. Mean prominence, which encompasses more information than a mere ‘yes’ or ‘no’ by considering the degree of relevance of the category, was used in analysis. SAS statistical software, version 9.2 (SAS Institute Inc., USA), was used.

#### Outcome variables

Anticipated OCV acceptance for free and at the low, medium and high prices were dichotomized into outcome variables denoting anticipated OCV acceptance or non-acceptance.

#### Logistic regression analysis and meta-analytic techniques

First, for each setting, univariable logistic regressions were done to identify sociocultural and sociodemographic determinants of anticipated OCV acceptance. Anticipated OCV acceptance at each price level served as outcome variables.

As a next step, meta-analytic techniques were employed to combine and compare associations between OCV acceptance and explanatory variables in the three settings. Meta-analysis refers to statistical methods for combining and contrasting results from two or more separate studies in order to identify patterns through increased statistical power and improved precision [26]. In this analysis, sociocultural and sociodemographic variables with consistent combined estimates (*p* < 0.1) and lacking heterogeneity (*p* > 0.1) were selected. Sociocultural variables were individually adjusted for sociodemographic variables from this selection. A fixed-effects meta-analysis of the adjusted estimates was done, and results are presented in forest plots, generated using STATA, version 10.1 (StatCorp LP, TX, USA). Figures 2 and 3 in this report display multiple forest plots, and each is an individual model. It was ensured that none of the models were over-fitted. Variables whose association with anticipated OCV acceptance showed heterogeneity at a level of *p* < 0.1 between the settings and a significant association in at least one of the settings (*p* < 0.05) are presented in tables. These variables were not meta-analysed owing to significant heterogeneity, but nevertheless provide valuable information regarding features of OCV acceptance unique to each setting.

Qualitative data were used to help explain quantitative associations. Narratives were written down in the respective local languages during the interview. They were then translated into English and typed in a word processor software using a pre-coded structure that reflected interview items. Transcripts of narratives were imported into MAXQDA 10 (Verbi software, Germany) and were coded using a deductive approach. Numeric explanatory and outcome variables were also imported into the MAXQDA data set, enabling selective retrieval of narratives based on identified quantitative relationships.

### Ethics statement

Ethical approval was obtained from the Ministry of Health Ethics Committee of Zanzibar for the study conducted in Zanzibar, Kenya Medical Research Institute for the study in W-Kenya and from the University of Kinshasa for the study conducted in SE-DRC. Furthermore, ethical approval was obtained for all three studies from the WHO Research Ethics Review Committee. All participants provided written informed consent before they were interviewed.

## Results

Data from a total of 1095 respondents were analysed—360 from SE-DRC, 379 from W-Kenya and 356 from Zanzibar. Approximately half the respondents in each of the settings were women. Mean age of respondents was similar in all three settings: 32.8 years in W-Kenya, 35.5 years in Zanzibar and 38.5 years in SE-DRC. Average household size was highest in Zanzibar, followed by SE-DRC and lowest in W-Kenya, with 6.8, 6.2 and 4.5 persons per household, respectively. A reliable and dependable income was reported by 35.3 % in SE-DRC, 47.8 % in W-Kenya and 55.9 % in Zanzibar. In W-Kenya and SE-DRC, 87.8 and 87.5 %, respectively, reported completing primary or secondary school education, while in Zanzibar 68.6 % had completed primary or secondary school. Most respondents in W-Kenya and SE-DRC reported Christianity as their religion, while in Zanzibar respondents were predominantly Muslim. The most commonly reported occupation in all three settings was agriculture (30.6 % in Zanzibar and SE-DRC, 26.6 % in W-Kenya). Further details on sample characteristics have been presented elsewhere [15, 17, 27].

### Anticipated OCV acceptance

**Fig. 1:**
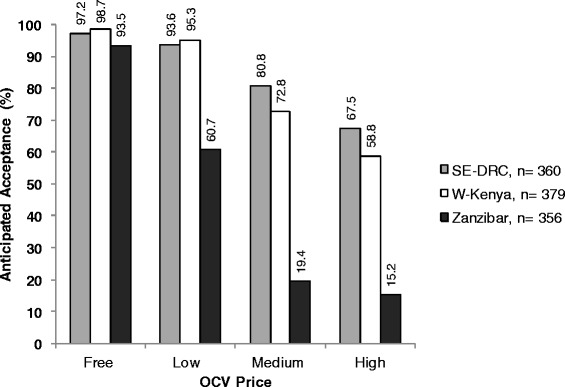
Anticipated oral cholera vaccine acceptance rates in three African settings at different price levels. OCV prices were stated to respondents in the local currency which was approximately equal to USD 1 (low price), USD 4–5 (medium price) and USD 8–11 (high price). Y-axis denotes percentage of respondents who provided an affirmative response when asked whether they would be likely to purchase the vaccine at the stated price. OCV: Oral cholera vaccine; SE-DRC: Southeastern Democratic Republic of Congo; W-Kenya: Western Kenya

High anticipated OCV acceptance rates (>93 %) were found in all settings when offered for free (Fig. 1), and acceptance decreased with increasing price. W-Kenya had the highest number of respondents willing to take the low-price and no-cost vaccine, while SE-DRC had the greatest number willing to purchase the medium-price and high-price OCVs. Zanzibar had the lowest anticipated OCV acceptance at all prices.

### Common determinants of OCV acceptance across all three settings

Near universal (>95 %) anticipated acceptance reported for the no-cost and low price OCV at some sites, made it unnecessary to further consider determinants at these price-levels. The medium price (USD 4–5) model approximates the cost for a full-course of Shanchol™ (USD 1.85 per dose [28, 29] = USD 3.70 for 2 doses). The high price (USD 8–10) model reflects the cost of Shanchol along with other programmatic and indirect costs. The high price also crudely approximates the market price for Dukoral™ (USD 5.25 per dose [28]), which was used for mass vaccination in Zanzibar.

#### Sociodemographic determinants

**Fig. 2:**
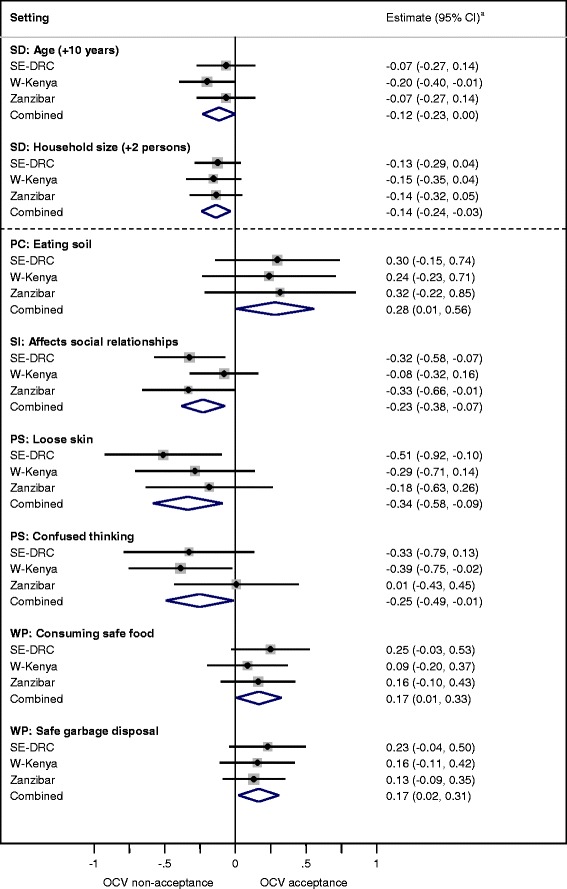
Sociocultural determinants of anticipated OCV acceptance at USD 4–5 (medium price) common to all settings. Forest plots depict the influence of sociodemographic and sociocultural variables on anticipated oral cholera vaccine acceptance at the medium price (USD 4–5) in three African settings. The weight of the study from each setting is represented by the area of the box whose centre represents the point estimate of effect from that study. The combined summary estimate of all three studies is represented by the centre of the diamond figure whose left and right extremes represent the corresponding confidence interval. ^a^ Logistic regression coefficient with 95 % confidence interval. Estimates have been adjusted for significant sociodemographic features (age, education, household size and occupation). SD: Sociodemographics; PC: Perceived causes of cholera; SI: Social impact of cholera; PS: Physical symptoms identified for cholera; WP: Ways to prevent cholera; OCV: Oral cholera vaccine; SE-DRC: Southeastern Democratic Republic of Congo; W-Kenya: Western Kenya

**Fig. 3:**
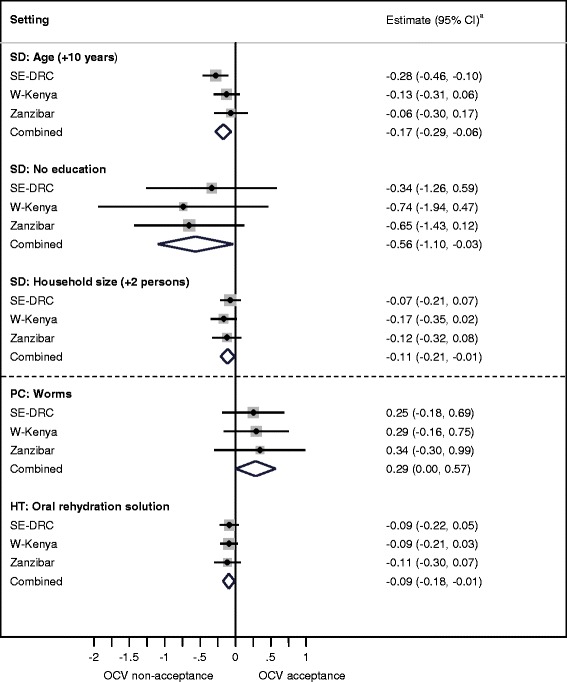
Sociocultural determinants of anticipated OCV acceptance at USD 8–11 (high price) common to all settings. Forest plots depict the influence of sociodemographic and sociocultural variables on anticipated oral cholera vaccine acceptance at the high price (USD 8–11) in three African settings. The weight of the study from each setting is represented by the area of the box whose centre represents the point estimate of effect from that study. The combined summary estimate of all three studies is represented by the centre of the diamond figure whose left and right extremes represent the corresponding confidence interval. ^a^ Logistic regression coefficient with 95 % confidence interval. Estimates have been adjusted for significant sociodemographic features (age, education, household size, sex and occupation). SD: Sociodemographics; PC: Perceived causes of cholera; HT: Home-based treatment, anticipated use of oral rehydration solution as a first-step at home in treating cholera; OCV: Oral cholera vaccine; SE-DRC: Southeastern Democratic Republic of Congo; W-Kenya: Western Kenya

Similar sociodemographic variables were significantly associated with anticipated OCV acceptance at the medium price of USD 4–5 (Fig. 2) and high price of USD 8–11 (Fig. 3). Increasing age and living in a larger household were associated with decreasing willingness to accept an OCV. Lack of education was a predictor of OCV non-acceptance at the high price. It was marginally significant at the medium price (*p* = 0.06, not represented in Fig. 2).

#### Sociocultural determinants

Sociocultural variables associated with anticipated OCV acceptance at the medium price were distinct from the high price. Only two were significantly associated at the high price, compared to six at the medium price. The following sociocultural determinants were common to all settings:

Attention to garbage disposal and consumption of safe food as measures to prevent cholera were predictors of OCV acceptance (Fig. 2).

Those identifying worms (Fig. 3) or the cultural practice of eating soil (Fig. 2) as causes of cholera were more likely to accept OCVs. Narrative accounts related the practice of eating soil to the fact that it is unclean, even though the practice may be culturally acceptable, especially for women. Eating unhygienic substances produced worms in the stomach. Respondents explained that they were trapped in an unclean environment, and though aware of the importance of hygiene, they felt there was little they could do to prevent cholera.

Knowledge of ORS for home-treatment of cholera was negatively associated with OCV acceptance at the high price (Fig. 3).

Identification of physical symptoms of dehydration such as loose skin and confused thinking were negatively associated with OCV acceptance (Fig. 2).

#### Social vulnerability

Respondents with more prominent concern about the effects of cholera on social relationships with others were less likely to anticipate purchasing OCV in all settings. The association was clearest in SE-DRC (Fig. 2).

### Setting-specific determinants of OCV acceptance

Sociocultural features of vaccine acceptance that were significantly heterogeneous across the three settings at the medium and high prices are presented in Tables 1 and 2.

**Table 1:** Sociocultural features of OCV acceptance heterogeneous across the three settings at USD 4–5 (medium price)

Features^a^	Heterogeneity *p*-value	Setting-specific estimates (95 % CI)^b^		
		SE-DRC	W-Kenya	Zanzibar
Vulnerability: Poor perceived more vulnerable	0.048	**1.38 (0.18, 2.58)**	−0.24 (−0.71, 0.23)	−0.06 (−0.67, 0.55)
Stigma: Others make patient feel ashamed	0.079	0.08 (−0.18, 0.33)	−0.12 (−0.33, 0.08)	**0.24 (0.00, 0.48)**
Physical symptom: Loss of appetite	0.069	−0.01 (−0.27, 0.25)	**−0.54 (−0.90, −0.17)**	−0.13 (−0.58, 0.31)
Physical symptom: Unconsciousness	0.032	**−0.26 (−0.47, −0.05)**	−0.03 (−0.28, 0.22)	0.12 (−0.07, 0.30)
Perceived cause: Witchcraft	0.048	0.24 (−0.11, 0.59)	0.28 (−0.50, 1.06)	**−0.60 (−1.19, −0.01)** ^**c**^
Regular, dependable income	0.002	−0.21 (−0.75, 0.34)	**1.02 (0.53, 1.50)**	**0.98 (0.39, 1.56)** ^**c**^
Married	0.018	−0.25 (−0.96, 0.46)	0.24 (−0.22, 0.70)	**1.37 (0.49, 2.24)** ^**c**^
Rural vs. urban site	0.049	0.11 (−0.42, 0.63)	**−0.63 (−1.09, −0.17)**	0.12 (−0.40, 0.65)

Sociocultural and sociodemographic features that are heterogeneously associated with oral cholera vaccine acceptance across three endemic African settings at the medium price of USD 4–5

Figures in bold represent associations with *p* < 0.05 for individual settings

*SE-DRC* Southeastern Democratic Republic of Congo; *W-Kenya* Western Kenya

^a^Variables with heterogeneity (*p*-value < 0.1) and a significant estimate (*p* < 0.05) in at least one of the settings are presented. Each row represents an association of the variable with anticipated OCV acceptance and each row is not adjusted for other variables in the table

^b^Setting-specific logistic regression coefficient with 95 % confidence interval

^c^Individual estimates for Zanzibar have been presented in Schaetti et al. [16]

**Table 2:** Sociocultural features of OCV acceptance heterogeneous across the three settings at USD 8–11 (high price)

Features^a^	Heterogeneity *p*-value	Setting-specific estimates (95 % CI)^b^		
		SE-DRC	W-Kenya	Zanzibar
Emotional impact: Sadness, anxiety, worry	0.046	**0.18 (0.03, 0.34)**	−0.14 (−0.35, 0.07)	0.14 (−0.13, 0.40)
Social impact: Fear of infecting others	0.009	**−0.33 (−0.57, −0.08)**	0.02 (−0.18, 0.22)	0.23 (−0.04, 0.50)^c^
Social impact: Interference with work/daily activities	0.064	**−0.31 (−0.52, −0.10)**	−0.04 (−0.19, 0.11)	0.01 (−0.19, 0.22)
Physical symptom: Pus in stool	0.033	−0.18 (−0.54, 0.18)	0.08 (−0.24, 0.41)	**0.75 (0.15, 1.36)** ^**c**^
Physical symptom: Nausea	0.098	**−0.50 (−0.89, −0.1)**	−0.23 (−0.65, 0.19)	0.31 (−0.31, 0.93)
Home treatment: Prayers	0.008	**0.38 (0.13, 0.63)**	**0.39 (0.09, 0.69)**	−0.24 (−0.58, 0.11)^c^
Regular, dependable income	<0.001	−0.31 (−0.77, 0.14)	**0.92 (0.49, 1.34)**	**0.94 (0.29, 1.59)** ^**c**^
Married	0.006	−0.45 (−1.05, 0.15)	0.18 (−0.24, 0.60)	**1.51 (0.46, 2.56)** ^**c**^
Rural vs. urban site	0.002	−0.03 (−0.47, 0.42)	**−0.67 (−1.08, −0.25)**	**0.64 (0.04, 1.23)** ^**c**^

Sociocultural and sociodemographic features that are heterogeneously associated with oral cholera vaccine acceptance across three endemic African settings at the high price of USD 8–11

Figures in bold represent associations with *p* < 0.05 for individual settings

*SE-DRC* Southeastern Democratic Republic of Congo; *W-Kenya* Western Kenya

^a^Variables with heterogeneity (*p*-value < 0.1) and a significant estimate (*p* < 0.05) in at least one of the settings are presented. Each row represents an association of the variable with anticipated OCV acceptance and each row is not adjusted for other variables in the table

^b^Setting-specific logistic regression coefficient with 95 % confidence interval

^c^Individual estimates for Zanzibar have been presented in Schaetti et al. [16]

#### SE-DRC

In SE-DRC, additional aspects of social vulnerability were apparent. ‘Fear of infecting others’ and ‘interference of cholera with work and daily activities’ were negatively associated with high-price OCV acceptance. These issues indicate a link between social vulnerability and lack of confidence in ability to pay for an OCV in SE-DRC. Psychological and personal emotional impact of cholera with reference to sadness and anxiety, on the other hand, was positively associated with OCV acceptance at the high price.

#### Zanzibar

Acknowledging the social disapproval of others in response to cholera was positively associated with acceptance of the medium-price vaccine in Zanzibar. Another cultural meaning of cholera, however—witchcraft as a perceived cause—was associated with non-acceptance of the medium-price OCV. Prayer was significantly associated with high-price OCV acceptance at both SE-DRC and W-Kenya, but not Zanzibar.

#### W-Kenya

Reporting a regular and dependable household income was positively associated with OCV acceptance in W-Kenya and Zanzibar. Narratives in W-Kenya included repeated community requests for a no-cost vaccine to provide access to everyone [15]. In W-Kenya, acceptance was less in the rural site for both medium- and high-priced OCVs.

## Discussion

Experience with OCV in vaccination campaigns has been steadily increasing [30]. To the best of our knowledge, this analysis is the first review of common and distinctive sociocultural determinants of anticipated OCV acceptance across multiple settings in Africa. Comparable research methods enabled a systematic meta-analytic approach. The findings identified patterns that would be unapparent in the individual studies, such as the identification of relevant determinants in all three populations. For example, the finding that knowledge of ORS for home-treatment of cholera was negatively associated with OCV acceptance at the high price was a unique finding from this meta-analysis, and it was not apparent (significant) from any of the individual country-specific studies. Our analysis is based on a systematic comparison of the three data sets, rather than a simple comparison of summary findings reported in the three published papers. The quantitative associations, derived and presented through forest plots, show how priority symptoms, perceived causes and options for help-seeking may influence OCV acceptance positively or negatively across different populations. Some factors have common effects across populations and others are setting-specific, indicating the value of local study to enable locally effective vaccine action. Although our methods are not a traditional meta-analysis, use of meta-analytic techniques highlight key sociocultural determinants common to three African settings and the importance of studying them.

Although anticipated acceptance may not perfectly reflect actual acceptance, observed priority for OCVs indicate that these communities desire benefits from such vaccination initiatives. The finding that fewer determinants of anticipated acceptance were identified for the high priced vaccine (two), compared with the medium price (six), clearly shows that increased cost imposes an economic barrier making other features of acceptance and demand irrelevant.

Paradoxically, SE-DRC has the greatest number willing to purchase the medium-price and high-price OCVs. People in W-Kenya and Zanzibar are economically better off as seen from gross domestic product per capita [31] and self-reported reliability of income among study respondents [18]. The seeming contradiction of greatest willingness to purchase OCVs among those with least economic resources may be explained by the serious trouble caused by cholera in SE-DRC, where public health facilities are often inaccessible or non-functional. Another point worth noting is that vaccines in SE-DRC are usually provided for free. The ability to pay is often overestimated when the scenario is hypothetical and respondents do not have to actually make the payment from their own pockets [32, 33]. The finding indicates community priority for a desired vaccine, rather than capacity to pay or prospects for effective uptake at the high price. Zanzibar has the lowest anticipated OCV acceptance at all prices. This may be an unintended consequence of a more accessible and effective public health system there compared to the other settings. Cholera camps instituted during an outbreak are accessible to most of the population who anticipate a fairly rapid response from local authorities [16]. Hence, the priority to pay for a vaccine may be reduced when timely life-saving treatment is assumed to be readily available compared with SE-DRC, where such confidence in lacking.

Findings suggest that when vaccine price is high, motivation to purchase OCV appears low among those with knowledge of feasible treatment options such as ORS. Vaccination and ORS seem to be competing interventions in the public mind. Zwisler et al. [34] found substantial satisfaction with ORS in treating diarrhoea among caregivers in Kenya and likely re-use of ORS in treatment if it had ever been used before. The marginal value of an OCV that users consider costly may be more limited in areas where ORS is well-known and widely used. Furthermore, priority for treatment may be valued more highly than prevention.

All study respondents were adults, and anticipated OCV acceptance was higher among younger adults. Lack of education in our study was associated with OCV non-acceptance. Other studies report a significant positive association between education and cholera-related knowledge [35]. Youth and better educated community residents may be a resource for vaccination campaigns to mobilize for community awareness of the benefits of vaccines.

Household size imposes economic constraints; more mouths to feed leaves less money available for other expenses, even if desired, including vaccines. OCVs are especially important for larger households which are more likely to be crowded and burdened by limited sanitation. Sharing a latrine with many households is a reported risk factor for cholera in Kenya [36]. Economic limitations affecting the most vulnerable segments of the community with the least resources highlights the priority of making OCVs available without cost to users. If provided at a low price, incentives or discounts for larger families may increase vaccine uptake.

Contrary to expectations, knowledge of dehydration symptoms decreased the priority of OCVs for prevention. Symptoms of dehydration, which are clearly related to cholera for health professionals, do not seem to be core features of a vaccine-preventable formulation of cholera in the community. Symptoms of dehydration may be linked in local perceptions to other forms of diarrhoea making a “cholera” vaccine less relevant. Although most respondents in the three studies identified the illness of the vignette as cholera or its local language equivalent (>85 %) [15, 17, 27], its link to dehydration appears less well understood.

In SE-DRC, social and economic vulnerability are interrelated, and both may constrain access to vaccines for those who may need it most. In Zanzibar, cholera-related stigma appears to motivate OCV acceptance, presumably to avoid stigma. However, vaccine acceptance was impeded by local magico-religious ideas, possibly reflecting a conflict between public health and interests of local healers.

In Zanzibar, religious influences appear less enabling for OCV acceptance. Although no active resistance from religious leaders is foreseen in Zanzibar, engaging religious leaders for vaccine action in all settings is important to build alliances and pre-empt opposition that may affect uptake [37], as indicated by notable opposition to polio vaccines in Nigeria [9, 38]. On the other hand, prayer and religious influences of a predominantly Christian population in SE-DRC and W-Kenya may promote vaccine use.

In W-Kenya, lower anticipated acceptance at the rural compared to the urban site, may result from urban–rural income disparities in W-Kenya, which was the only setting where fewer rural than urban respondents reported reliable and dependable incomes (*p* < 0.001) [15]. Access and uptake would appear to be more sensitive there to the effect of price. In Zanzibar, however, rural respondents were more likely to accept the high-price OCV. These findings suggest that urban–rural differences in vaccine acceptance may vary across settings based on local conditions and priorities.

### Limitations and strengths

Data for this analysis were collected between 2008 and 2010 during a period of high cholera burden in all three settings. More recent WHO data indicate a persisting cholera burden in DRC (33,661 cases). In United Republic of Tanzania however, fewer cases (286) were reported, and in Kenya no cases of cholera were reported in 2012 [39]. The decline in cholera cases in Kenya was attributed to effectiveness of water, sanitation and hygiene (WASH) interventions by public health officials (Personal communication, Public Health Officials in Kisumu and Siaya. Conversations during a dissemination activity conducted by the research team at study sites in Western Kenya, 2013). More recently, however, rapid spread of cholera has been noted in Kenya with 3301 cases reported between December 2014 and May 2015 [40]. High CFR of 2 % have been noted country-wide with some counties reporting CFR as high as 7.6 %. Zanzibar appears to have benefitted from the OCV campaign that was undertaken there. Thus, priority for use of OCVs changes with the change in cholera epidemiology. While OCVs are not indicated in settings with no more cholera, findings of this study and community priority for preventing cholera remain relevant not only for consideration in future outbreaks but also for implementation of other WASH interventions. Findings and the approach presented in this comparative analysis are relevant for settings where there is a clear rationale for use of OCVs.

Although detailed narrative data were collected during interviews that lasted approximately one hour, sample size was a limitation in the quantitative analysis. Due to practical constraints, between 356 and 379 interviews could be conducted in each setting. The sample size allowed for limited adjustment and all explanatory variables could not be adjusted against each other.

In addition to the benefits of increased power through meta-analytic techniques that enable identification of determinants that may not be apparent in individual studies, a major strength of this cross-setting analysis is the identification of sociocultural determinants of OCV acceptance that are generalizable across multiple populations. Local study is necessary to understand nuances of vaccine acceptance or hesitancy that are influenced by local culture and context. However, questions of generalizability of findings to other settings often arise. By considering sociocultural determinants of anticipated OCV acceptance from local study, but common across three distinct populations, broader and more generalizable determinants have been distilled that are useful in guiding policy for wider use and implementation of OCVs by national and global policy makers and public health professionals.

Recent developments towards setting up of a global stockpile for OCVs is based on the assumption that it would be used by the populations it is given to. The approach presented in this study, which makes it possible to distinguish common and setting-specific sociocultural factors affecting OCV acceptance is especially timely in view of opportunities arising for effective use of OCVs enabled by the development of a global stockpile. It is also relevant for enhancing coverage of other vaccines through consideration of community determinants.

The community perspective is relevant not only for OCVs but also for consideration of community-related determinants of vaccine effectiveness, such as hesitancy, demand and access. A rapid assessment of such community interests can be expected to contribute to the effectiveness of vaccine action. Based on experience with this approach for community assessment, development and validation of rapid assessment tools are needed to demonstrate the usefulness of the approach for enhancing uptake in programme settings.

## Conclusion

The identified sociocultural determinants of OCV acceptance show that cost constraints are an essential consideration for effective use of OCVs. Paradoxically, awareness and appreciation of the value of treatment with ORS was associated with less enthusiasm for the OCV, and the setting with best prospects for treatment showed least interest in prevention with OCV. Findings indicated community interest and demand for cholera interventions. The absence of major sociocultural barriers to vaccination with OCVs suggest good prospects for translating vaccine efficacy into programme effectiveness in epidemic and endemic settings where vaccines have a role to play in control and elimination of cholera.

## Abbreviations

EMIC: Explanatory Model Interview Catalogue
OCVs: Oral cholera vaccines
ORS: Oral rehydration solution
SE-DRC: Southeastern Democratic Republic of Congo
USD: United States dollars
WHO: World Health Organization
W-Kenya: Western Kenya
